# Insect adhesion on rough surfaces: analysis of adhesive contact of smooth and hairy pads on transparent microstructured substrates

**DOI:** 10.1098/rsif.2014.0499

**Published:** 2014-09-06

**Authors:** Yanmin Zhou, Adam Robinson, Ullrich Steiner, Walter Federle

**Affiliations:** 1 Department of Zoology, University of Cambridge, Downing Street, Cambridge CB2 3EJ, UK; 2 Department of Physics, Nanoscience Centre, Cavendish Laboratory, J.J. Thomson Avenue, Cambridge CB3 0HE, UK

**Keywords:** adhesion, tribology, biomechanics, photolithography, surface roughness, contact mechanics

## Abstract

Insect climbing footpads are able to adhere to rough surfaces, but the details of this capability are still unclear. To overcome experimental limitations of randomly rough, opaque surfaces, we fabricated transparent test substrates containing square arrays of 1.4 µm diameter pillars, with variable height (0.5 and 1.4 µm) and spacing (from 3 to 22 µm). Smooth pads of cockroaches (*Nauphoeta cinerea*) made partial contact (limited to the tops of the structures) for the two densest arrays of tall pillars, but full contact (touching the substrate in between pillars) for larger spacings. The transition from partial to full contact was accompanied by a sharp increase in shear forces. Tests on hairy pads of dock beetles (*Gastrophysa viridula*) showed that setae adhered between pillars for larger spacings, but pads were equally unable to make full contact on the densest arrays. The beetles' shear forces similarly decreased for denser arrays, but also for short pillars and with a more gradual transition. These observations can be explained by simple contact models derived for soft uniform materials (smooth pads) or thin flat plates (hairy-pad spatulae). Our results show that microstructured substrates are powerful tools to reveal adaptations of natural adhesives for rough surfaces.

## 1. Introduction

Many insects are capable of producing adhesion on natural substrates, which usually possess some degree of surface roughness [1]. Insect claws can interlock with asperities larger than approximately the diameter of the claw tips [2,3], and adhesive pads have to be used when surface protrusions are too small for the claws to interlock with [4,5].

Insects have evolved two distinct types of adhesive systems, hairy and smooth pads [6], which can adjust to surface roughness using different mechanisms. The hairy adhesives, as found in beetles [7] and flies [8,9], are arrays of fibres tipped with thin, flat spatulae. The fibres are able to bend on a length scale of tens of micrometres and thereby conform to larger surface features, whereas the thin (less than 200 nm) and flexible spatula tips can bend to follow smaller-scale roughness [10].

By contrast, the smooth pads as found in ants, cockroaches and stick insects [5,11–14] possess a soft, specialized adhesive cuticle which can also deform and adjust to surface roughness. The pads' thick procuticle contains cuticular rods oriented approximately perpendicular to the surface, branching out into finer fibres towards the thin epicuticle. The internal fibrous structure of smooth pads may be important for their deformability [14,15], for the shear-induced lateral increase in contact area [16] or for the efficient transfer of tensile forces, but its detailed function is still unclear. For both smooth and hairy pads, small length scales of surface roughness may be further compensated by the adhesive fluid secreted from the pads, which provides capillary and viscous adhesion and appears to be present in all insect adhesive pads [17].

Despite these adaptations, it has been observed that surface microroughness asperity size less than *ca* 5 µm can strongly reduce insect attachment and climbing ability [2,18–20]. Even for soft solids, surface roughness can produce a loss of real contact area (where intimate contact between the contact bodies is achieved), and thereby strongly reduce adhesion [21–23]. This adhesion-reducing effect of surface roughness is exploited by many plants, some of which have surfaces covered with microrough epicuticular wax crystals to dispel insects [24], probably enhanced by the exfoliation of crystals and contamination of the adhesive structures [25,26]. The fine cuticular folds found on many plant surfaces may serve a similar function [27].

So far, studies on biological adhesives have focused mainly on smooth substrates. The current, poor quantitative understanding of animal adhesion on rough surfaces may be based on two important experimental limitations: (i) the complex and irregular surface topography of natural substrates makes it difficult to distinguish the critical length scales that affect the insects' attachment ability and (ii) the non-transparency of these substrates does not allow direct observations of the adhesive contact.

Here, we address these limitations by using microstructured, transparent substrates as simple models for rough surfaces. This approach allows us not only to test the effects of specific length scales on adhesion, but also to visualize the adhesive contact zone. We fabricated microstructured, transparent surfaces containing square arrays of pillars with different heights and spacings, and used them as substrates to test the attachment performance of smooth and hairy pads.

With our experiments, we address the following questions:

(1) Under what conditions are smooth and hairy pads able to make full contact?
(2) How does surface topography influence the shear forces of insect pads?
(3) Is the deformation of smooth and hairy pads consistent with simple indentation models?

## 2. Methods

### 2.1. Fabrication of microstructured substrates

Microstructured transparent substrates with standardized topographies were fabricated using photolithography and nanoimprinting. A ‘master’ mould was first produced in negative photoresist (MicroChem, SU-8 2000.5 or SU-8 2002) via ultraviolet light through a lithography shadow mask. SU-8 2000.5 or SU-8 2002 photoresist (viscosities 2.49 and 7.5 cSt, respectively) was spin coated onto a clean silicon wafer for 30 s at a velocity of 2000 r.p.m. The heights of the features were determined by the thickness of the resulting photoresist layers (0.5 or 1.4 µm). The in-plane dimensions of features followed those on the lithography mask. This method was used to fabricate ‘master’ arrays of cylindrical pillars on silicon substrates. In order to make the master non-adhesive, it was placed with 100 µl of perfluorodecyltrichlorosilane in an evacuated desiccator and left overnight. The master was then coated with freshly mixed polydimethylsiloxane (PDMS; Sylgard 184, Dow Corning), degassed in a desiccator to remove air from the base of the features and cured in an oven for 24 h at 65°C to produce a soft inverse ‘mould’. A freshly mixed low-viscosity epoxy (Robnor Resins, PX672H/NC) was then poured onto the PDMS mould and again degassed before a clean glass coverslip (18 × 18 × 0.1 mm) was placed on top of the epoxy. After 24 h curing at room temperature, the mould was removed gently to leave a precise replica of the master in transparent epoxy on the glass coverslip. The cured epoxy has a stiffness of *ca* 1.8 GPa (measured using bending tests), so that deformations of the substrate are negligible under our experimental conditions.

**Figure 1.**
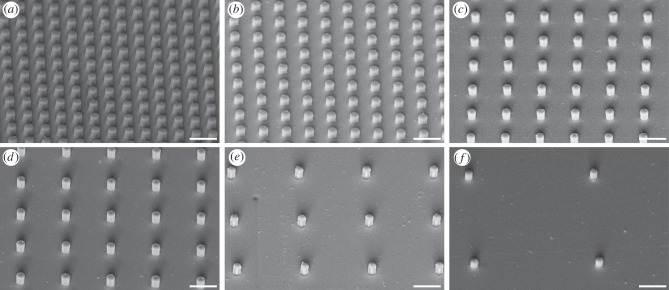
SEM micrographs of microstructured transparent epoxy substrates with squared arrays of cylindrical pillars of 1.4 µm in height and diameter. The centre-to-centre spacing is (*a*) 3, (*b*) 4, (*c*) 6, (*d*) 8, (*e*) 12 and (*f*) 22 μm. Scale bar, 5 µm.

Using this method, substrates with well-defined, cylindrical pillars of different spacing and height were produced. All pillars had a diameter of 1.4 µm and were arranged in square arrays with centre-to-centre spacing of 3, 4, 6, 8, 12 or 22 µm. One complete set was produced with a pillar height of 0.5 µm (termed ‘short’ pillars throughout this paper), and the other set was produced with a pillar height of 1.4 µm (termed ‘tall’ pillars throughout this paper; figure 1).

### 2.2. Scanning electron microscopy

Scanning electron microscopy (SEM) was used to characterize the microstructured substrates. Microstructured substrates were mounted on SEM stubs, and sputter-coated with a 5 nm thick layer of gold to prevent charging, using a Quorum Technologies K575X turbo-pump sputter coater at 65 mA for 15 s. Samples were viewed using a Leo Gemini 1530VP field emission gun scanning electron microscope with a beam voltage of 5 kV.

### 2.3. Study animals

Adult cockroaches (*Nauphoeta cinerea*; body mass 444 ± 20 mg; mean ± s.e.m., *n* = 20) and male dock beetles (*Gastrophysa viridula*; body mass 9.31 ± 0.34 mg; mean ± s.e.m., *n* = 20) were taken from laboratory colonies kept in plastic containers. Cockroaches were kept at 24°C and fed on dog food; dock beetles were kept at 20°C and fed on fresh dock leaves. For light microscopy and force measurements, cockroaches were immobilized by mounting them on their back using Parafilm tape on a microscope slide glued on a glass tube. A hind leg with trimmed claw tips was fixed on the dorsal side with vinyl polysiloxane impression material (Elite HD + light body, Zhermack, Badia Polesine, Italy) to a piece of soldering wire that was attached to the microscope slide. Dock beetles were immobilized by embedding them on their back in Blu-Tack and Parafilm tape mounted on a glass tube; Blu-Tack was also used to isolate the hind leg. The last tarsal segment and the claws were bent over and fixed in the Blu-Tack to expose the distal pad on the third tarsal segment and to prevent the claws from touching surfaces during experiments.

### 2.4. Visualization of adhesive contact area

Reflected-light microscopy was used to characterize the contact of insect adhesive pads on transparent microstructured surfaces. The pad contact zone was viewed through the transparent surface with a Leica DMR-HC upright microscope using monochromatic epi-illumination (546 nm) at 20× or 100× magnification. Images were taken using a QICAM 8-bit monochrome camera.

### 2.5. Single-pad force measurements

Following previous studies [17,28], a custom-made set-up was used to perform force measurements on single adhesive pads of live insects.

A transparent epoxy substrate (smooth or microstructured) mounted on a glass coverslip was attached to the end of a two-dimensional bending beam mounted on a three-dimensional motor positioning stage (M-126PD, C-843, Physik Instrumente, Germany). The bending beam was moved by the motor stage to position the substrate to make contact with the isolated adhesive pad of a live insect. The pad contact area was visualized using a stereomicroscope with a coaxial illumination. Contact areas were recorded at 10 Hz using an externally triggered Redlake PCI 1000 B/W video camera mounted on a stereo microscope.

The two-dimensional force transducer consisted of a metal beam (34.5 × 4.2 × 0.2 mm) folded three times to produce two axes at a right angle to each other. Two 350 Ω foil strain gauges (1-LY13-3/350, Vichay) were glued on each axis of the beam to form half bridges. The spring constant of the two-dimensional force transducer used was 40.89 N m^−1^ in the normal direction and 7.55 N m^−1^ in the lateral direction. The voltage output was amplified (ME-Meβsysteme, Henningsdorf, Germany) and recorded to a computer via an I/O board (PCI-6035E, National Instruments, USA) with a sampling frequency of 20 Hz.

A custom-made LabVIEW (National Instruments) program controlled the movements of the motor stage, recorded friction and normal forces and triggered the video camera to record the contact area synchronously. The program includes a force feedback mechanism (frequency: 20 Hz) allowing the normal force to be maintained constant during a friction force measurement. Contact area and friction force recordings were analysed using Matlab (The Mathworks, USA). For both smooth and hairy pads, projected contact areas were measured manually by drawing a polygon around the outer rim of the pad contact zone.

Before each experiment, the substrate was brought into contact with the insect adhesive pad for 15 s with a normal force of 0.4 mN. The substrate was then moved for 20 s across the insect pad, away from the insect (corresponding to a horizontal pull of the leg towards the body), with a constant normal force of 0.4 mN and a dragging velocity of 0.1 mm s^−1^. After the motor stopped pulling, the pad was held in contact with the same normal force for another 10 s before the substrate was pulled off perpendicularly at a velocity of 0.5 mm s^−1^. The peak friction force during the pulling and the corresponding projected contact area were analysed to calculate shear stress (maximum friction force per projected contact area).

For both beetles and cockroaches, the normal force feedback was kept at 0.4 mN during the slide. As both insects had similar projected contact areas (beetles: 54 685 ± 2099 μm^2^, cockroaches: 49 376 ± 2365 μm^2^; means ± s.e.m., *n* = 20 each), this force resulted in similar mean load stresses (beetles: 7.3 kPa, cockroaches: 8.1 kPa).

## 3. Results

### 3.1. Contact zone morphology of insect pads on microstructured substrates

#### 3.1.1. Smooth pads

**Figure 2.**
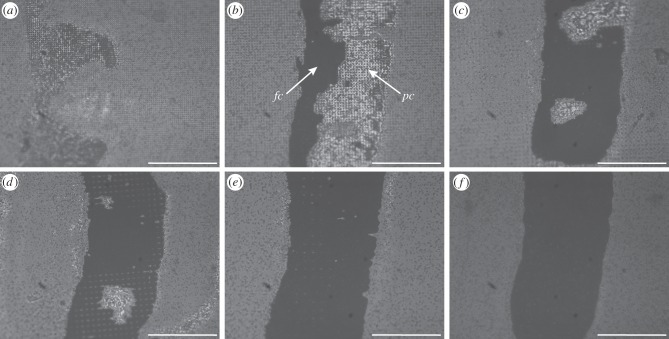
Static contact images of the cockroach arolium (*Nauphoeta cinerea*) on squared arrays of ‘tall’ pillars (1.4 µm in diameter and height), visualized by reflected-light illumination. The centre-to-centre spacing is (*a*) 3, (*b*) 4, (*c*) 6, (*d*) 8, (*e*) 12 and (*f*) 22 μm. *fc*, full contact; *pc*, partial contact. Scale bar, 100 μm.

When the smooth footpads of cockroaches were brought into contact with tall (1.4 μm high) pillar arrays, they made either partial or full contact (figure 2). Partial contact was observed for pillar arrays with 3 μm spacing (figure 2*a*), where the arolium made contact with the tops of the pillars only. Full contact occurred on the substrates with pillar spacings of 4 μm or larger (figure 2*b–f*), where the arolium deformed sufficiently to make contact to both the tops of the pillars and the substrate between them. Full and partial contact could occur side by side on the same pad; this was observed in particular for the substrate with 4 μm spacing (figure 2*b*). Pads making such ‘transitional’ contact did not show any consistent location of the zones in full contact within the contact zone, indicating that load pressures were approximately constant within the contact zone.

**Figure 3.**
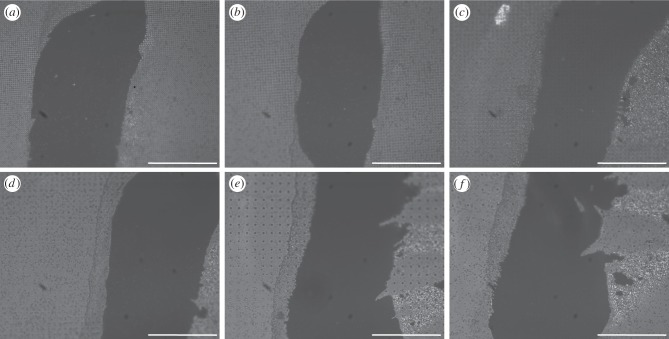
Static contact images of the cockroach arolium (*Nauphoeta cinerea*) on squared arrays of ‘short’ pillars (1.4 µm in diameter and 0.5 µm in height), visualized by reflected-light illumination. The centre-to-centre spacing is (*a*) 3, (*b*) 4, (*c*) 6, (*d*) 8, (*e*) 12 and (*f*) 22 μm. Scale bar, 100 μm.

Unlike the situation on tall pillars, cockroach arolia made full contact on all of the substrates with short (0.5 μm high) pillars (figure 3).

#### 3.1.2. Hairy pads

**Figure 4.**
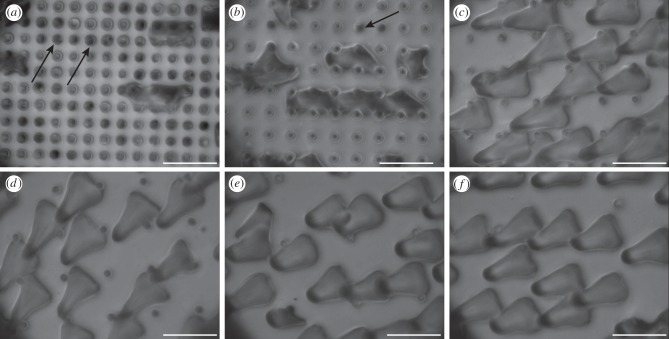
Static contact images of the spatula tips of the beetle pad (*Gastrophysa viridula*) on squared arrays of ‘tall’ pillars (1.4 µm in diameter and height), visualized by reflected-light illumination. The centre-to-centre spacing is (*a*) 3, (*b*) 4, (*c*) 6, (*d*) 8, (*e*) 12 and (*f*) 22 μm. Arrows in (*a*,*b*) indicate pillar tops touched by spatulae in partial contact. Scale bar, 10 μm.

**Figure 5.**
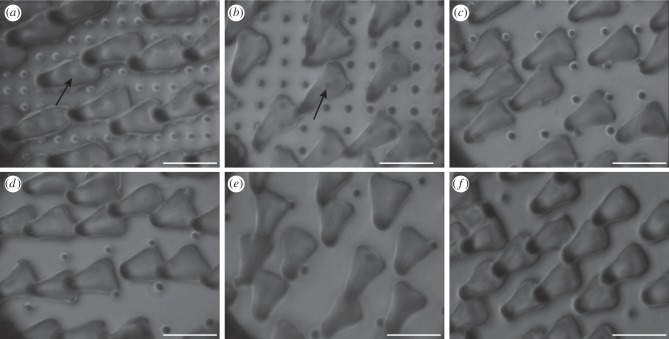
Static contact images of the spatula tips of the beetle pad (*Gastrophysa viridula*) on squared arrays of ‘short’ pillars (1.4 µm in diameter and 0.5 µm in height), visualized by reflected-light illumination. The centre-to-centre spacing is (*a*) 3, (*b*) 4, (*c*) 6, (*d*) 8, (*e*) 12 and (*f*) 22 μm. Arrows indicate spatula tips standing on pillars in (*a,b*). Scale bar, 10 μm.

As a consequence of their geometrical structure, the beetles' adhesive hairs could ‘bypass’ the pillar microstructures for spacings larger than 4 µm, because the setal spatula tips were able to make contact with the smooth substrate between the pillars (figures 4*c*–*f* and 5*c*–*f*). Only for the two densest pillar arrays were the spacings too narrow for the spatulae. On the substrates with dense tall pillars, some of the spatulae were in partial contact, touching only the tops of the pillars, whereas others appeared to bend or fold to fit into the gaps between pillars (so that the contacts were no longer spatula-shaped but followed the square lattice; figure 4*a,b*). The formation of such contacts on dense arrays was possibly helped by liquid secretion.

**Figure 6.**
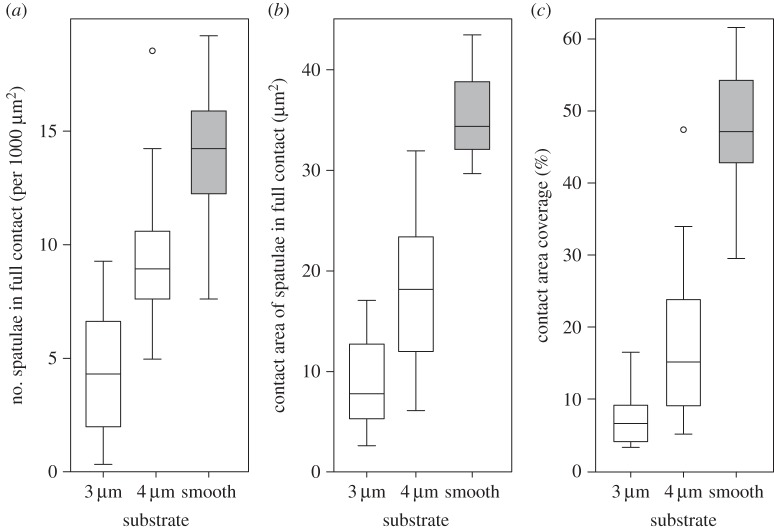
Contact of spatulae of beetle pad (*Gastrophysa viridula*) on the two densest squared arrays of tall pillars (3 and 4 µm spacing, 1.4 µm in diameter and height) in comparison with smooth surfaces. (*a*) Number of spatulae per 1000 µm^2^ that were in full contact, (*b*) contact area of individual spatulae in full contact (µm^2^) and (*c*) overall contact area coverage (%) of hair array. Centre lines and boxes represent the median within the 25th and 75th percentiles, whiskers show the 10th and 90th percentiles and circles indicate outliers.

On the two densest arrays of tall pillars, the number of hairs in full contact was strongly reduced in comparison with the smooth substrate (figure 6*a*; linear mixed-effect model with substrate as fixed, and beetle as random factor: *F*_2,48_ = 120.1, *p* < 0.001; no difference between the substrate with 6 µm spacing and the smooth surface: *F*_1,24_ = 0.013, *p* = 0.91). Moreover, individual spatulae that were in full contact had a significantly reduced contact area on the two densest substrates (figure 6*b*; linear mixed-effect model: *F*_2,48_ = 191.1, *p* < 0.001; again no difference between 6 µm spacing and smooth: *F*_1,24_ = 1.11, *p* = 0.30). The combination of both factors, i.e. partial contact of many setae and reduced contact area for the few setae in full contact, resulted in an overall reduced contact area on dense pillar arrays (figure 6*c*; linear mixed-effect model: *F*_2,48_ = 301.7, *p* < 0.001).

By contrast, no such deformation was observed on the dense arrays of short pillars. Here, spatulae appeared to be in full contact, touching both the tops of the pillars and the substrate between them (figure 5*a*,*b*). However, reflections from the dorsal side of the very thin spatulae lead to stray light and unfavourable optical conditions, making it difficult to distinguish unequivocally between full and partial contact.

### 3.2. Friction force of insect pads on microstructured substrates

On both smooth and microstructured surfaces, we observed a gradual transition from static contact to sliding, which was not associated with a decrease in friction force. Instead, friction forces increased when pads began to slide and usually kept increasing.

#### 3.2.1. Smooth pads

**Figure 7.**
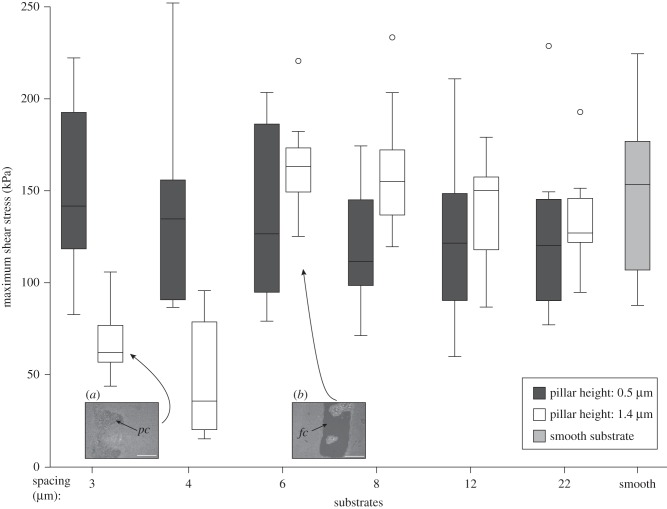
Maximum shear stress of smooth pads of cockroaches (*N. cinerea*) on microstructured and smooth substrates. The shear stress was smallest on the two densest tall (1.4 μm high) pillar arrays where pads made partial contact. Centre lines and boxes represent the median within the 25th and 75th percentiles, whiskers show the 10th and 90th percentiles and circles indicate outliers. *fc*, full contact; *pc*, partial contact. Scale bar, 100 μm.

The maximum shear stresses of cockroach pads sliding on the substrates with tall pillars were strongly reduced on the two densest arrays of pillars (with 3 and 4 μm spacing) compared with the other four tall-pillar substrates and the smooth surface (figure 7). Friction force and shear stress significantly decreased for denser pillar arrays (Page's *L*-test, *p* < 0.001; table 1). Pairwise comparisons showed that force and shear stress changed most strongly between the substrates with 4 and 6 µm spacing (Wilcoxon signed rank-sum tests, *p* < 0.01, all other comparisons *p* > 0.05; table 1).

**Table 1.** Statistical results for friction forces and shear stresses (force per projected contact area) for smooth pads (*N. cinerea*) and hairy pads (*G. viridula*). Page's *L*-test was used to identify changes in shear force/stress with increasing pillar spacing. When a trend was present, consecutive pillar spacings were compared using pairwise Wilcoxon signed rank-sum tests.

	pillar height (µm)	Page's *L*-test	pairwise Wilcoxon signed rank-sum tests for consecutive pillar spacings (µm)				
			3 versus 4	4 versus 6	6 versus 8	8 versus 12	12 versus 22
*Nauphoeta cinerea*							
friction force	0.5	*L*_6,10_ = 686 *p* > 0.05	—	—	—	—	—
shear stress	0.5	*L*_6,10_ = 706 *p* > 0.05	—	—	—	—	—
friction force	1.4	*L*_6,10_ = 845 *p* < 0.001	0.110	0.002	0.920	1.000	0.850
shear stress	1.4	*L*_6,10_ = 836 *p* < 0.001	0.084	0.002	0.920	0.130	0.850
*Gastrophysa viridula*							
friction force	0.5	*L*_6,11_ = 976 *p* < 0.001	0.007	0.007	0.067	0.067	0.320
shear stress	0.5	*L*_6,11_ = 981 *p* < 0.001	0.083	0.007	0.042	0.054	0.410
friction force	1.4	*L*_6,15_ = 34.5 *p* < 0.001	0.190	<0.001	0.140	0.055	0.980
shear stress	1.4	*L*_6,15_ = 32.2 *p* < 0.001	0.035	<0.001	0.140	0.150	0.800

By contrast, no such reduction of the maximum force and shear stress was observed for the substrates with short pillars (figure 7; Page's *L*-test, *p* > 0.05; table 1).

#### 3.2.2. Hairy pads

**Figure 8.**
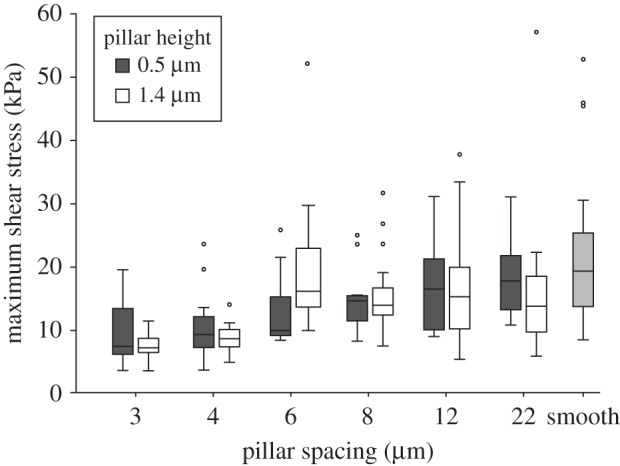
Maximum shear stress of the hairy pads of beetles (*G. viridula*) on microstructured and smooth substrates. Shear stress was reduced on dense arrays, both for tall and short pillars. Centre lines and boxes represent the median within the 25th and 75th percentiles, whiskers show the 10th and 90th percentiles and circles indicate outliers.

Similar to the smooth pads of cockroaches, the maximum force and shear stress of the beetles' hairy pads were reduced on denser tall-pillar arrays (Page's *L*-test, *p* < 0.001; table 1). However, this effect was also observed for arrays with short pillars (figure 8; Page's *L*-test, *p* < 0.001; table 1).

For both the short and tall pillar substrates, the maximum force and shear stress again changed most strongly between the substrates with 4 and 6 µm spacing (Wilcoxon signed rank-sum tests, *p* < 0.01; table 1). The changes in the maximum force and shear stress on the short pillars appeared less stepwise and more gradual than for the tall pillars and smooth pads (figure 8 and table 1).

## 4. Discussion

In this study, transparent epoxy substrates with well-defined microstructures were used to study the interaction of insect adhesive structures with rough substrates. We find a transition from full contact to partial contact and a consistent reduction in shear forces for dense arrays of tall pillars in insects with smooth and hairy pads. While a loss of adhesion on rough substrates has been reported in previous studies on smooth and hairy footpads of insects [2,19,20,24,27,29,30], our contact images reveal in detail how contact area is affected by surface roughness.

In order to understand the pads' performance on the microstructured substrates, we will discuss the conditions leading to full or partial contact separately for smooth and hairy pads.

### 4.1. Smooth pads

We compare our observations with a simple model for the contact between a block of isotropic material and a substrate with stiff cylindrical pillars (see appendix A). As the arolium cuticle in *N. cinerea* cockroaches is much thicker than the height of the pillar substrates we used (thickness 14.5–61 µm [11]), this simplification appears justified. From a balance between the adhesive energy gained and the elastic energy penalty for deformation (ignoring normal forces), it can be predicted that a smooth pad should make full contact to a substrate patterned with cylindrical pillars if [31]4.1
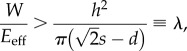
where *W* is the work of adhesion of the pad, *E*_eff_ is the effective elastic modulus of the pad, *h* is the pillar height, *d* is the pillar diameter, *s* is the centre-to-centre spacing between pillars and *λ* is a summarizing topography parameter with dimensions of length.

**Figure 9.**
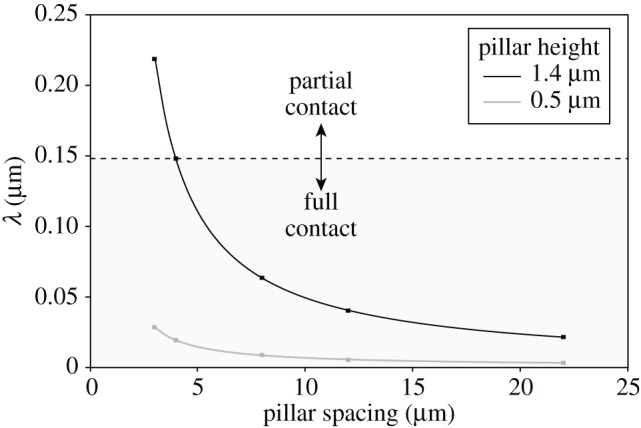
Plot of the topography parameter *λ* of the smooth pad contact model as a function of pillar spacing. Dashed line indicates the transition from partial to full contact observed for cockroach pads at *λ* ≈ 0.15 μm; partial contact was observed above this line. Squares indicate values of *λ* calculated for the tested microstructured substrates.

In our experiments, the transition from partial to full contact occurred on the arrays with tall (1.4 μm) pillars and 4 μm spacing, corresponding to *λ* ≈ 0.15 μm. Smaller values of *λ* indicate that it is easier for the pad to make full contact, whereas larger values predict incomplete (partial) contact, where the pad touches only the tops of the pillars (figure 9). All our present results from cockroach pads can be explained by a transition from partial to full contact for *λ* ≈ 0.15 μm. Assuming a work of adhesion *W* = 40 mN m^−1^ and *λ* = 0.15 μm, the pad's effective elastic modulus can be estimated to be approximately 270 kPa, consistent with measurements for smooth pads of bushcrickets and stick insects [14,15].

Our simple model does not consider the effect of normal forces. Higher normal forces would shift the balance between elastic and adhesive energy more towards adhesion and full contact.

### 4.2. Hairy pads

Two different regimes were observed for the contact of hairy pads with pillar arrays. When the spacing of asperities was larger than the diameter of the spatula tip (*ca* 4.3 µm; figures 4 and 5), the spatula tips were able to make full contact in between the pillars and slide on the substrate. However, if the surface projections were denser (3 and 4 µm spacing), the spatula tips had to bend to reach the substrate in between the pillars, possibly aided by the capillary action of the adhesive secretion.

**Figure 10.**
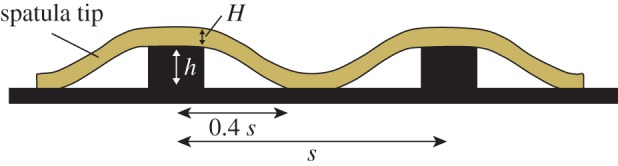
Model for a thin spatula tip in contact with a pillar array substrate. *h* is the pillar height, *s* is the centre-to-centre spacing between pillars and *H* is the thickness of the spatula. (Online version in colour.)

In order to understand the interaction of the hair tips with the microstructures in the ‘spatula bending’ regime, we modelled a single pillar in contact with a spatula as a thin circular plate or membrane with supported edges subjected to a concentrated load in its centre (figure 10 and see appendix A). From a balance between the adhesive energy and elastic energy (again ignoring normal forces), we find that the spatula tip should make full contact to a substrate patterned with cylindrical pillars if4.2
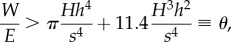
where *W* is the work of adhesion of the spatula tip, *E* is the elastic modulus of the spatula tip, *h* is the pillar height, *H* is the thickness of the spatula tip, *s* is the centre-to-centre spacing between pillars and *θ* is a summarizing topography parameter with dimensions of length.

**Figure 11.**
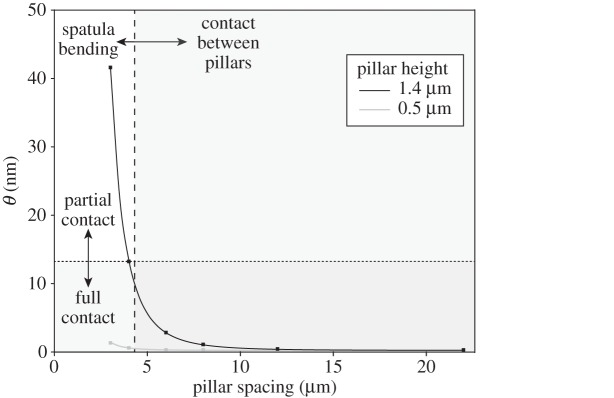
Plot of the topography parameter *θ* of the spatula contact model as a function of pillar spacing. Dashed line indicates the width of the beetles' spatula tip (≈4.3 µm); setae can slide between pillars to the right of this line. Dotted line indicates the value of *θ* above which spatulae in partial contact were observed. Squares indicate individual values of *θ* calculated from corresponding microstructured substrates.

Analogous to the above analysis for smooth pads, one can calculate a transition value of *θ* below which the spatulae should make full contact. If *W* is estimated to be 40 mN m^−1^, and *E* is estimated to be 1.2 MPa (measured for the setae of the ladybird beetle *Coccinella septempunctata* [32]), full contact would occur if the parameter *θ* is smaller than 33 nm. With an estimate for the spatula thickness *H* of 250 nm [33], full contact would be expected for all but the densest tall pillar substrate (*θ* = 41.6 nm; all other substrates *θ* ≤ 13.2 nm; figure 11). As we observed partial contact at least for our densest tall pillar substrate, the elastic modulus for spatulae of *G. viridula* may be similar to that measured for *C. septempunctata.* Thus, our results support the finding by Peisker *et al*. [32] that the spatulae have a distinctly softer cuticle than the seta stalks (5–16 GPa [34]).

Both above models for smooth and hairy pads show that the balance between adhesive and elastic energy is dependent on the size (length scale) of asperities. While the adhesive energy scales with area, the elastic energy for deformation scales with volume. As a consequence, adhesion dominates for small length scales (small *λ*, *θ*), whereas the elastic energy penalty is more important for larger surface features (large *λ*, *θ*), consistent with conclusions of previous studies [35–38]. This scaling relationship explains why most plant surfaces that are slippery for insects possess microroughness at the length scale between 0.1 and 5 µm. The claw tips of most insects are too blunt to grip on asperities in this size range, but the features are still large enough to prevent strong adhesion. Reducing insect adhesion with even smaller surface asperities may be impracticable for plants, because according to equations (4.1) and (4.2), such asperities would need to be produced with higher and higher aspect ratios to reach the topography parameter (*λ* or *θ*) required to prevent full contact.

### 4.3. Frictional performance

Both smooth and hairy pads showed a clear reduction in shear forces for the two densest substrates with tall pillars. This reduction in shear force can be explained by the observed loss of full contact and reduction in adhesive contact area on these substrates.

Friction forces on smooth substrates for soft polymers such as rubber have been found to depend linearly on the area of contact [39,40]. However, while for cockroaches the friction force on the densest array of tall pillars (3 µm centre-to-centre spacing and 1.4 µm pillar diameter and height) was reduced to 37% of the friction force on the smooth substrate, the area on the tops of the pillars on this substrate represented only 17%. The values for beetles show the same trend (friction force reduction to 40%, contact area reduction to 14%). Thus, the force reduction for smooth and hairy pads was smaller than predicted if shear forces were directly proportional to real contact area.

This mismatch may be explained firstly by the contact of smooth pads or spatulae to the lateral walls of the pillars, thereby increasing the adhesive contact area. A simple calculation shows that if pads made contact to the walls over the uppermost 0.4 µm of each pillar, the contact area would correspond to 37% of a smooth substrate, potentially resolving the above discrepancy. A similarly increased real contact area may explain that under certain conditions, adhesives on rough surfaces can achieve forces exceeding those on smooth substrates [41]. This would occur if the additional gain in adhesive energy outweighs the elastic penalty for making full contact.

Second, higher friction forces on rough surfaces could also be caused by oscillating deformations of the pad as it slides across the microstructured substrate. This effect has been described for the friction of rubber [37,42]. At least in smooth pads, the deformations may lead to energy dissipation, thereby increasing friction forces. It is still unclear how this component of friction depends on surface topography and on other test conditions such as normal force and sliding velocity. Nevertheless, the friction enhancement by wall contacts or rubber friction in our experiments was not so high that friction forces exceeded those on the smooth substrate.

While a visible loss of contact area can explain the reduced shear forces on the tall pillar arrays, the beetles' reduced shear forces on dense arrays of short pillars cannot be directly explained with our contact zone images, which indicated full contact of all spatulae. However, even when pads are in full contact without any visible air gaps between the pillars, it is likely that there is a ring-shaped zone around each pillar in which the pad or spatula is deflected away from the surface and the gap is filled with adhesive secretion. These zones may contribute only weakly to shear forces, thereby reducing forces on denser arrays even when pillars are short and allow full contact. It is unclear why we did not find the same trend for the smooth pads of *N. cinerea*. In our study, the shear stresses measured for *N. cinerea* cockroaches generally exceeded those of *G. viridula*. It is unlikely that this represents a general difference between smooth and hairy pads, as we had previously found comparable levels of shear stress for smooth pads (*Carausius morosus* stick insects) and hairy pads (*G. viridula*) under different experimental conditions [28]; the detailed factors underlying the difference in this study are still unclear. Overall, however, surface roughness had surprisingly similar effects for smooth and hairy adhesive pads.

Our present results for pillar arrays of two heights and constant diameter can be explained by assuming that smooth pads or spatula tips of hairy pads each consist of dry, isotropic, linearly elastic materials. However, it is likely that adhesive pads have more complex properties. First, both smooth and hairy adhesive pads inject fluid secretions into the contact zone which improve the pads' ability to make contact to rough surfaces by increasing adhesive contact area [17,43,44]. Second, at least, the cuticle of smooth pads has viscoelastic properties [15], leading to creep and a time-dependent ability to conform to substrates. Third, smooth pad cuticle has a fibrous inner structure and is likely to exhibit anisotropy. Thus, the pads' ability to conform to the substrate may depend on the direction of loading and on the applied shear force. Fourth, insect pads are unlikely to have the same stiffness at all length scales. Localized indentation measurements using atomic force microscopy in stick insects revealed that their smooth pads exhibit a stiffness gradient, with the outermost, 300 nm thick epicuticle being significantly softer than the inner cuticular layers [14]. Although adhesion should become inherently stronger at smaller length scales (see above), such a stiffness gradient may be advantageous as it can help to make contact on substrates which become rougher at smaller length scales [14].

Our study shows that microstructured, transparent substrates with a well-defined topography provide a powerful tool to study the adaptations of natural adhesives for making contact on rough surfaces. While these substrates have simpler surface profiles than natural rough surfaces, they allow visualization of the pad's contact area at different length scales, and systematic tests of the pad's performance under different experimental conditions.

## Data accessibility

All data are available from the Dryad digital repository (http://doi.org/10.5061/dryad.k8d1f).

## Appendix A

### A.1. Contact model for smooth pads on pillar substrates

In order to describe the contact between smooth insect pads and a substrate with cylindrical pillars, we use an approximate model that makes several simplifying assumptions. First, we consider the pad cuticle to be a block of a linearly elastic, isotropic material. Second, we assume that the pressure is uniform across the pad's contact zone, i.e. the load on each pillar is assumed to be the same. As pads with mixed full and partial contact did not show any consistent position of the full contact within the contact zone (unlike in comparable experiments for PDMS spheres in contact with PDMS microstructures [45,46]), the latter assumption appears justified. Third, we assume that full contact (i.e. contact with the bottom of the pillar array) will be made once the pad is able to fill out a ‘cavity’ within the square element formed by four pillars. The depth of this cavity is *h* and its width is approximately  where *s* is the centre-to-centre spacing between pillars, *h* is the height and *d* is the diameter of the pillars.

Following McMeeking *et al*. [31], the insect pad will spontaneously fill out the cavity between four pillars if

A1

where *λ* is a summarizing topography parameter with dimensions of length.

It is predicted that this topography parameter, *λ*, determines the contact regime of a smooth pad with a substrate with cylindrical pillars. The smooth pad will make full contact with the substrate if , whereas a larger *λ* predicts partial contact between the pad and the substrate.

### A.2. Contact model for hairy pads (spatulae) on pillar substrates

Even when surface features are smaller than the size of a spatula tip, adhesive hairs may still make full contact by bending and stretching of the thin spatula. To estimate how the spatula is deformed by the pillars, we modelled the spatula zone interacting with one pillar as a thin, circular plate (or membrane) of radius *a* with simply supported edges, subjected to a normal force at its centre. A closed zone of full contact of the spatula around each pillar will be achieved if *a* < *s*/2, i.e. if the plate radius is less than half the centre-to-centre spacing between pillars (figure 10).

The deformation of a circular plate is dominated by bending for thicker plates and small displacements, but by stretching for very thin membranes and larger strains. According to Komaragiri *et al*. [47], a single dimensionless parameter can be used to determine whether bending or stretching dominates, given by

A2

where *F* is the normal force in the centre of the membrane, *H* is the membrane's thickness, *E* is the elastic modulus and *v* is Poisson's ratio. While bending dominates for small values of *ψ*, stretching is more important for large *ψ*, i.e. for large loads, thin films and compliant material. For the conditions of our experiment, 0 < *ψ* < *ca* 2000, indicating that both bending and membrane solutions are applicable (for small and large strains, respectively). As accurate models are not available, we model the force during spatula indentation as the sum of a bending term (dominating for small strains) and a membrane term (dominating for large strains).

#### A.2.1. Circular plate bending model

The deflection at the centre of a loaded thin plate is [48]

A3

where *w*_max_ is the maximum deflection of the plate, *c* is the radius of the loaded area, *F* is the normal force applied on the spatula tip, *v* is Poisson's ratio of the spatula tip (estimated as *v* = 0.3) and *D* is the flexural rigidity of the spatula tip. Assuming that the central point of load application is small, we neglect terms containing *c*^2^, yielding

A4

The flexural rigidity of a plate is [48]

A5

where *H* is the thickness of the spatula and *E* is its elastic modulus. By combining equations (A 4) and (A 5), we obtain

A6

Hence, the normal force *F* is

A7

#### A.2.2. Membrane model

The classic solution by Schwerin [49] for a point load acting in the centre of a membrane without pre-stretch is

A8

This point-load solution is valid for relatively small *d*/2*a* ratios (i.e. when contact radius is much smaller than membrane diameter); for larger ratios, more complex solutions would be required [50]. Exact solutions also depend on the material's Poisson ratio, and on the shape of the indenter [47,51–53], but the abovementioned simple model is sufficient for deriving an approximate scaling relationship.

In order to make contact with the substrate between the pillars, the maximum deflection of the spatula needs to be as large as the height of the pillars, implying

A9

where *h* is the height of the pillars. Hence, the normal force *F* is

A10

#### A.2.3. Combined plate and membrane model

As the force results both from bending and stretching of the spatula, we approximate the force by the sum of the circular plate and membrane expressions (equations (A 7) and (A 10)):

A11

The elastic energy *U*_E_ that it costs the spatula tip to deform around each pillar can be calculated as

A12

The adhesion energy *U*_A_ that the spatula tip gains by making contact with the substrate between the pillars is

A13

where *W* is the work of adhesion.

Minimizing total energy *U*_T_ with respect to contact area radius *a*:

A14

Full contact will be favoured if *U*_T_(*a**) < 0, which gives

A15

where *θ* is a summarizing topography parameter with dimensions of length. The energy minimum occurs for

A16

which is the radius shown in figure 10.

According to the model, this topography parameter *θ* determines whether spatula tips can make full contact with an array of pillars. Full contact may be achieved for *θ* < *W*/*E*, whereas a larger *θ* would result in partial contact between the spatula tip and the substrate.
